# Changes in codon-pair bias of human immunodeficiency virus type 1 have profound effects on virus replication in cell culture

**DOI:** 10.1186/1742-4690-10-78

**Published:** 2013-07-25

**Authors:** Gloria Martrus, Maria Nevot, Cristina Andres, Bonaventura Clotet, Miguel Angel Martinez

**Affiliations:** 1 Fundació irsiCaixa, Hospital Universitari Germans Trias i Pujol, Universitat Autònoma de Barcelona (UAB), Badalona 08916, Spain

**Keywords:** Codon-pair bias, HIV, Attenuation, Evolution

## Abstract

**Background:**

Human immunodeficiency virus type 1 (HIV-1) has a biased nucleotide composition different from human genes. This raises the question of how evolution has chosen the nucleotide sequence of HIV-1 that is observed today, or to what extent the actual encoding contributes to virus replication capacity, evolvability and pathogenesis. Here, we applied the previously described synthetic attenuated virus engineering (SAVE) approach to HIV-1.

**Results:**

Using synonymous codon pairs, we rationally recoded and codon pair–optimized and deoptimized different moieties of the HIV-1 gag and pol genes. Deoptimized viruses had significantly lower viral replication capacity in MT-4 and peripheral blood mononuclear cells (PBMCs). Varying degrees of ex vivo attenuation were obtained, depending upon both the specific deoptimized region and the number of deoptimized codons. A protease optimized virus carrying 38 synonymous mutations was not attenuated and displayed a replication capacity similar to that of the wild-type virus in MT-4 cells and PBMCs. Although attenuation is based on several tens of nucleotide changes, deoptimized HIV-1 reverted to wild-type virulence after serial passages in MT-4 cells. Remarkably, no reversion was observed in the optimized virus.

**Conclusion:**

These data demonstrate that SAVE is a useful strategy to phenotypically affect the replicative properties of HIV-1.

## Background

Given the structure of the genetic code synonymous codons will yield distinct amino acid changes upon mutation. The extraordinarily large number of possible encodings in natural genes is to some extent restricted by two encoding biases referred to as codon bias [1,2] and codon pair bias [3]. The remarkable nucleotide composition of the human immunodeficiency virus type 1 (HIV-1) genome with an above average percentage of A nucleotides results in a codon composition that is different from that of the human genome [4]. Especially the more flexible third codon positions are preferentially occupied by A-nucleotides [5], which induces ribosome pausing and inefficient translation [6]. The bias of A-rich codons in HIV-1 is thought to be the action of host enzymes of the APOBEC3 family. On the contrary, it has been shown that the HIV-1 A-rich sequence is not merely an evolutionary artefact of enzyme-induced hypermutations, and that HIV-1 has adapted to rely on A-rich RNA sequences to support the synthesis of viral cDNA during reverse transcription [7]. Recent data have identified a novel antiviral mechanism within the innate immune response, in which human SLFN11 selectively inhibits viral protein synthesis in HIV-infected cells by means of codon-bias discrimination [8]. Synonymous substitutions optimizing for human cell expression reduces the antiviral activity of SLFN11 [8]. Synonymous mutation is thought to be selectively neutral. However, previous work demonstrated that synonymous substitutions can negatively affect the replication capacity of several RNA viruses [9], including HIV-1 [10].

Recently, a new approach, termed synthetic attenuated virus engineering (SAVE), was used to rationally design live attenuated poliovirus and influenza virus vaccines [3,11]. SAVE works by recoding and synthesizing the viral genome; the wild-type amino acid sequence is preserved, but the existing synonymous codons are rearranged to create a suboptimal arrangement of codon pairs [3]. Although the reasons are still not well understood, some codon pairs occur more frequently, and others less frequently, than expected [12]. The overall codon usage in a genome can differ dramatically among species, although less so among closely related species [13]. It is unclear why some codon pairs are under- or overrepresented. It has been suggested that structural features that regulate tRNA geometry within the ribosome may govern genomic codon pair patterns, driving enhanced translational fidelity and/or rate [14]. For example, as described by Coleman et al., the amino acid pair Ala-Glu is expected to be encoded by GCCGAA and GCAGAG approximately equally as often. However, the codon pair GCCGAA is strongly underrepresented and is used only one-seventh as often as GCAGAG [3].

SAVE was used previously to recode poliovirus and influenza virus genomes so that they include infrequently used codon pairs, which led to the generation of highly attenuated viruses [3,11]. Mice immunized with recoded attenuated polioviruses were found to have protective immunity when they were challenged with wild-type poliovirus [3]. The mechanism of attenuation is unclear, but it has been suggested that translation is affected [3,11]. Since codon-pair deoptimization is the result of tens, hundreds, or even thousands of nucleotide substitutions, it has been also suggested that reversion to virulence is unlikely to occur. Furthermore, attenuation may be fine tuned by adjusting the extent of codon-pair deoptimization [3].

In contrast to poliovirus and influenza virus, after retrotranscription of its genomic RNA into a proviral intermediate, HIV-1 (a retrovirus belonging to the Lentiviridae family) DNA is permanently integrated into the host cell genome and transcribed by the host RNA polymerase II [15]. Moreover, HIV-1 can initiate translation either by the classical cap-dependent mechanism or by internal recruitment of the ribosome through RNA domains called IRESs (internal ribosome entry sites) [16]. To explore whether recoding synonymous codon pairs will generate viruses with altered replication capacity, we redesigned different parts of the HIV-1 genome. To phenotypically modify the replicative properties of HIV-1, we recoded the gag and protease (contained within the pol gene) coding regions by introducing optimized or deoptimized codon pairs without altering the amino acid sequence. Gag and protease coding regions were chosen in order to explore proteins with different function, structural and enzymatic, respectively. When redesigned gag and protease fragments were recombined with HIV-1 infectious clones that lacked gag or protease, the resulting viruses showed significantly modified viral replication capacity in MT-4 cells and peripheral blood mononuclear cells (PBMCs).

## Results

### Construction of synthetically recoded HIV-1 with modified codon-pair bias

We recoded the HIV-1 protease and gag coding regions by introducing different codon pairs. The virus was recoded to use codon pairs that are underrepresented or overrepresented relative to the human genome by means of a previously described algorithm [3]. Only synonymous substitutions were introduced. Recoded segments had the same amino acid sequence as the wild-type segments, but different pairwise arrangements of synonymous codons. Codon bias and the folding free energy of the RNA were also controlled (Additional file 1: Table S1, Additional file 2: Figure S1). Recoded fragments were chemically synthesized, recombined with an infectious HIV-1 DNA clone in which protease or gag had been deleted and the resulting viral stocks sequenced. To avoid that recombination was prevented by the mutations, no substitutions were introduced in the recombination regions.

**Figure 1:**
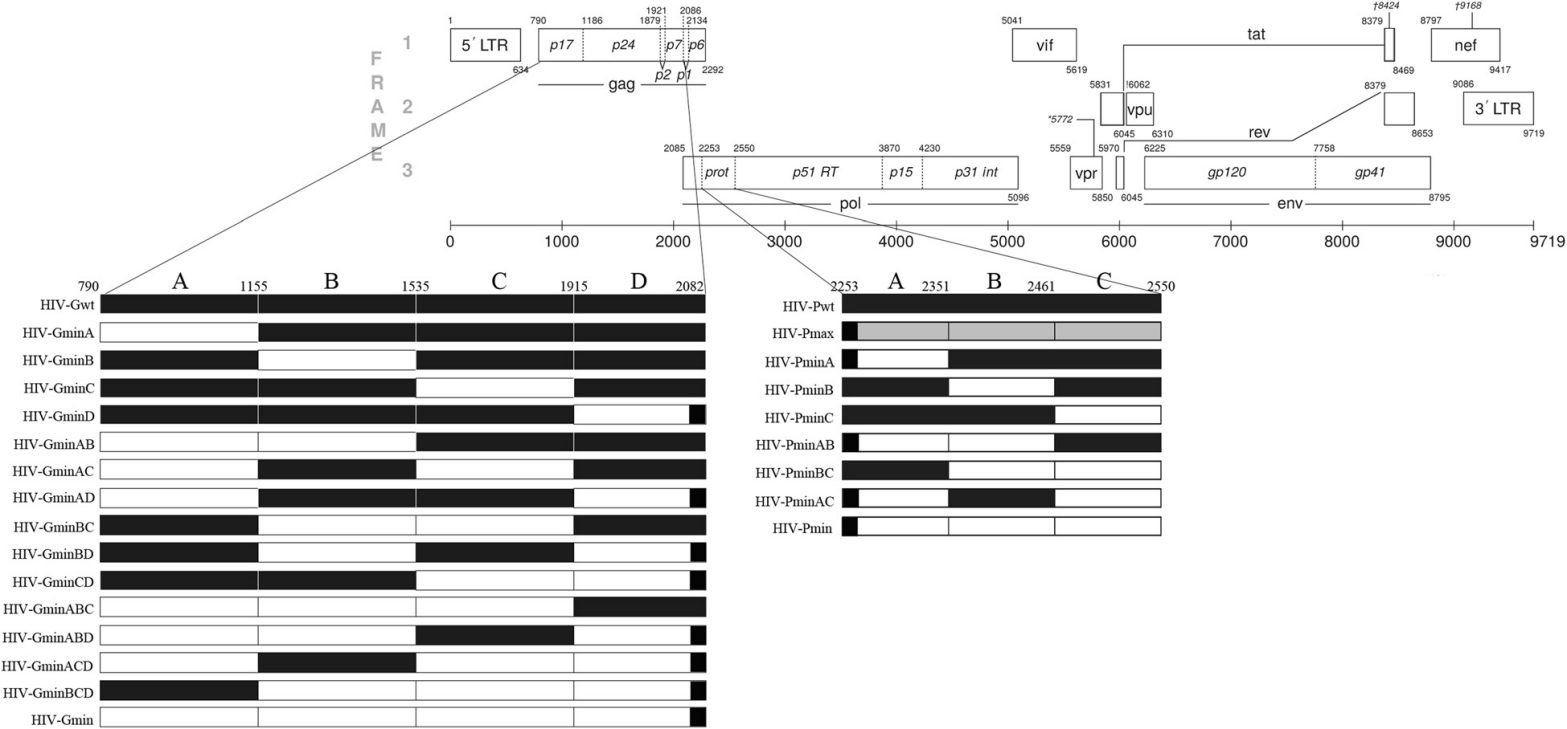
**Schematic representation of HIV-1 genome and structures of various chimeric, partly synthetic, recoded HIV-1 constructs.** Wild-type sequences are depicted in black, deoptimized sequences in white, and optimized sequences in grey.

**Figure 2:**
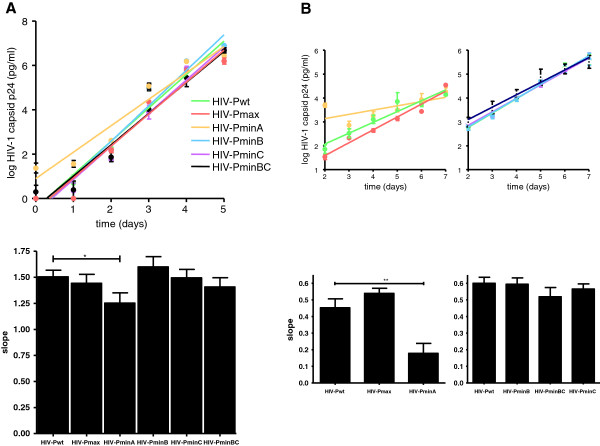
**Replication kinetic assay of HIV-1 recoded protease variants. (A)** MT-4 cells, **(B)** PBMCs. Production of the HIV-1 antigen p24 in culture supernatants was determined on days 0–5 (MT-4 cells) and on days 2–7 (PBMCs). For each virus, the slope of the plot provides an estimate of the viral replication capacity. The slope of the p24 antigen production of each virus after infection of MT-4 cells and PBMCs is shown by the bars. Comparisons between wild-type (HXB2) and mutant recoded viruses are shown. The significance of the difference between slopes was calculated with GraphPrism v. 4 software (unpaired *t*-test). Values represent the mean ± standard deviation (SD) from at least three independent experiments.

We first designed and constructed an entire deoptimized protease gene, HIV-Pmin, as well as an optimized protease gene, HIV-Pmax (Figure 1). Of the 297 nucleotides that encode the HIV-1 protease, HIV-Pmin and HIV-Pmax contain 57 and 38 synonymous substitutions, respectively (Table 1). HIV-Pmin has a codon pair–bias score (see Methods) much lower than that of human genes, while HIV-Pmax has a codon pair–bias score much higher than that of human genes (Table 1). The percentage of As in the HIV-Pmin sequence decreased from 0.36 to 0.30, and the percentage of Gs increased from 0.23 to 0.27. In contrast, the percentage of As in the HIV-Pmax sequence slightly increased to 0.38 and the percentage of Gs, 0.22, was almost identical to that of the wild-type sequence. After recombination of these two synthetic constructs with an HIV-1 infectious clone, no viable virus could be isolated from MT-4 cells transfected with HIV-Pmin, even after five blind passages. As expected, HIV-Pmax yielded a viable virus that was titrated and replicated. When the replication capacity of HIV-Pmax was assayed in MT-4 cells and PBMCs, HIV-Pmax was as fit as the wild-type virus in both cell types (Figure 2).

**Table 1:** Codon-pair bias scores of selected HIV-1 protease recoded synthetic constructs

**Protease**	**CPB score**	**Number of mutations**
HIV-Pwt	0.069	0
HIV-Pmax	0.254	38
HIV-PminA	−0.094	15
HIV-PminB	−0.094	22
HIV-PminC	−0.154	18
HIV-PminBC	−0.317	41
HIV-PminAB	−0.256	38
HIV-PminAC	−0.317	29
HIV-Pmin	−0.480	57

To obtain deoptimized viable virus, we reduced the number of underrepresented pair codons present in HIV-Pmin (Figure 1). These new variants, which have a different number of synonymous substitutions and different codon-pair scores (Table 1), yielded viruses with diverse ex vivo replication capabilities (Figure 2). HIV-PminB, HIV-PminC, and HIV-PminBC produced viable virus in MT-4 cells and PBMCs with a replication capacity that was indistinguishable from the wild-type virus (*p* = 0.2317, 0.8742, and 0.1984, respectively, for MT-4 cells and *p* = 0.8539, 0.2789 and 0.1014, respectively, for PBMCs). Growth competition experiments in MT-4 cells between the former variants and the wild-type virus confirmed that these deoptimized protease viruses had a replication capacity similar to that of the wild-type virus (data not shown). In contrast, HIV-PminA, with only 15 synonymous substitutions (Table 1), produced a virus that had its replication capacity highly reduced in PBMCs (*p* < 0.001) and MT-4 cells (*p* < 0.05) (Figure 2). Of note, all constructs except HIV-PminA had a similar infectivity/p24 antigen ratio compared to HIV-Pwt (see time 0 on Figure 2A and B). This result was also an indicator of the HIV-PminA attenuation. No viable virus was obtained from HIV-PminAB and HIV-PminAC constructs.

**Figure 3:**
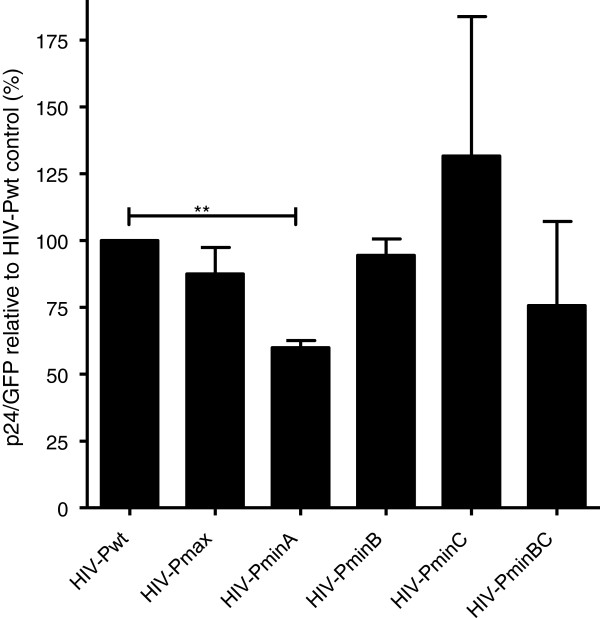
**Single-cycle infectivity assay of HIV-1 protease codon pair–recoded synthetic constructs.** HIV-1 protease codon pair–recoded synthetic constructs (Figures 1 and 2) were introduced into the GFP reporter HIV-1 infectious clone pNL4-3-deltaE-EGFP. The replication capacities of the different protease constructs are represented as a percentage relative to the wild-type HIV-1 HXB2 strain (100%). The relative replication capacity of the virus was determined by measuring the amount of p24 antigen. A reduction in the production of p24 protein in the culture transfected with a HIV-1 clone carrying a deoptimized protease was observed. The reduction in p24 expression was not accompanied by a reduction in the production of gag RNA (Table 3).

To investigate the possible effect of codon pair bias on translation, we used a GFP reporter HIV-1 single cycle infectious clone (pNL4-3-deltaE-EGFP). Because GFP expression depends on the vector transfection efficiency only and not on the viral replication capacity, normalization of GFP production after transfection can be used to quantify viral growth, viral transcription, and viral translation efficiency. HIV-1 protease recoded variants were used in these experiments. After cell vector transfection and GFP normalization, quantification of the production of viral protein p24 confirmed the lower replication capacity of the HIV-PminA deoptimized variant compared to wild-type virus (Figure 3). The reduction in p24 was not accompanied by a reduction in the production of gag RNA (Table 2), suggesting that deoptimized codon pairs reduced translation.

**Table 2:** Real-time quantitative RT-PCR analysis of HIV-1 gag (p24) and GFP gene RNAs after one cycle of viral replication

	**HIV-Pwt**	**HIV-Pmax**	**HIV-PminA**	**HIV-PminB**	**HIV-PminC**	**HIV-PminBC**
GFP Ct^a^	17.01 ± 0.05	17.22 ± 0.07	17.97 ± 0.04	17.15 ± 0.07	17.10 ± 0.14	17.14 ± 0.02
gag Ct	17.89 ± 0.39	17.40 ± 0.37	18.02 ± 0.36	17.36 ± 0.66	17.53 ± 0.14	17.86 ± 0.19
GAPDH Ct	16.46 ± 0.04	16.25 ± 0.23	16.52 ± 0.30	16.65 ± 0.62	16.49 ± 0.31	16.22 ± 0.04
2^(−ΔΔCt)^b^ gag/GADPH	1.00	1.22	0.95	1.66	1.32	0.91
2^(−ΔΔCt) gag/GFP	1.00	0.94	0.96	0.94	0.91	0.97

^a^Ct, threshold cycle.

^b^Livak formula for relative gene expression analysis.

**Figure 4:**
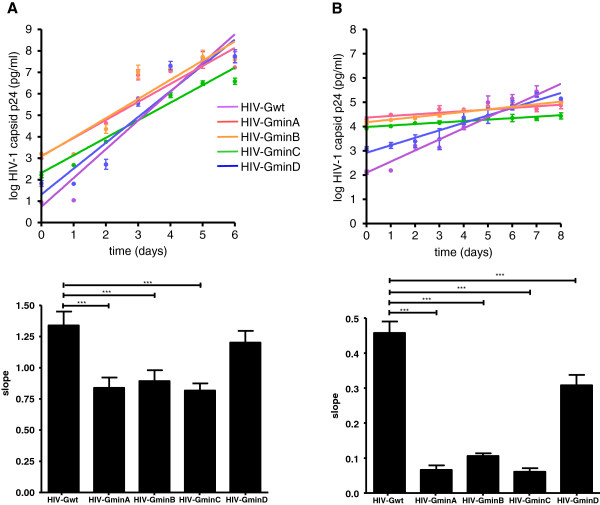
**Replication kinetic assay of HIV-1 recoded gag variants. (A)** MT-4 cells, **(B)** PBMCs. Production of the HIV-1 antigen p24 in culture supernatants was determined on 0–5 (MT-4 cells) and on days 2–7 (PBMCs). For each virus, the slope of the plot provides an estimate of the viral replication capacity. The slope of the p24 antigen production of each virus after infection of MT-4 cells and PBMCs is shown by the bars. Comparisons between wild-type (HXB2) and mutant recoded viruses are shown. The significance of the difference between slopes was calculated with GraphPrism v. 4 software (unpaired *t-*test). Values represent the mean ± SD from at least three independent experiments.

To explore how codon-pair deoptimization could affect other HIV-1 genomic regions, we deoptimized the gag coding region. Gag encodes the viral capsid proteins and therefore has a very different role in HIV-1 biology from that of virus protease. Three hundred and eight synonymous substitutions were introduced in the 1 503 nucleotides of gag. No substitutions were introduced in the gag-pol ribosomal slip site or in the p6 portion of gag, in order to avoid lethality and/or the introduction of nonsynonymous substitutions. Similar to the results obtained with the protease variants, HIV-Gmin (Figure 1) yielded no viable virus. Again, the number of underrepresented pair codons present in HIV-Gmin (Figure 1) was reduced. Only the constructs HIV-GminA, HIV-GminB, HIV-GminC, and HIV-GminD, which contain 118, 97, 93, and 34 synonymous substitutions, respectively (Table 3), produced viable viruses. Similar to deoptimized protease sequences, these four constructs had a lower percentage of As and an increased percentage of Gs (data not shown). Likewise, the HIV-GminA, HIV-GminB, HIV-GminC, and HIV-GminD constructs had a lower infectivity/p24 antigen ratio compared to HIV-Gwt (see time 0 on Figure 4A and B), suggesting that they were attenuated. Indeed, these four viable deoptimized viruses showed a highly significant attenuated phenotype in PBMCs (*p* < 0.001). HIV-GminA, HIV-GminB, and HIV-GminC were also highly attenuated in MT-4 cells (*p* < 0.001) (Figure 4).

**Table 3:** Codon pair bias scores of selected HIV-1 gag recoded synthetic constructs

**Gag**	**CPB Score**	**Number of mutations**
HIV-Gwt	0.035	0
HIV-GminA	−0.091	118
HIV-GminB	−0.077	97
HIV-GminC	−0.070	93
HIV-GminD	−0.011	34
HIV-GminAB	−0.203	215
HIV-GminAC	−0.193	211
HIV-GminAD	−0.133	152
HIV-GminBC	−0.183	190
HIV-GminBD	−0.123	131
HIV-GminCD	−0.113	127
HIV-GminABC	−0.305	308
HIV-GminABD	−0.242	249
HIV-GminACD	−0.235	245
HIV-GminBCD	−0.225	224
HIV-Gmin	−0.348	342

We next tested the susceptibility of recoded HIV-1 variants to antiretroviral compounds. The association between hypersusceptibility to protease inhibitors and low replication capacity of viruses isolated from chronically infected patients promoted us to test the susceptibility of recoded HIV-1 variants to antiretroviral compounds [17]. The IC50 of all viable gag and protease recoded variants to two NRTIs, AZT and TNF, and four protease inhibitors, NFV, DRV, ATV and APV, was calculated. No differences in drug susceptibility were found between wild-type virus and low or high fitness gag or protease recoded variants (Additional file 3: Table S2).

Overall, our results demonstrate that the codon pair recoding of HIV-1 gag or protease coding regions significantly modified HIV-1 replication capacity in MT-4 cells and PBMCs.

### Stability of attenuated phenotypes

**Figure 5:**
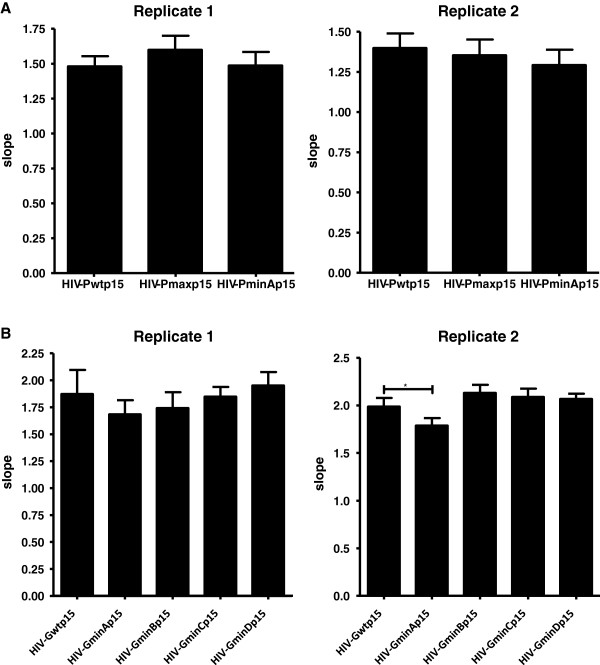
**Replication kinetic assay of HIV-1 recoded variants after 15 serial passages in MT-4 cells. (A)** Protease variants, **(B)** gag variants. Passages were started with a MOI, 0.0002. Two replicates were performed for each variant. Production of the HIV-1 antigen p24 in culture supernatants was determined on days 0–5. For each virus, the slope of the plot provides an estimate of the viral replication capacity. The slope of the p24 antigen production of each virus after infection of MT-4 cells is shown by the bars. Comparisons between wild-type (HXB2) and mutant recoded viruses are shown. The significance of the difference between slopes was calculated with GraphPrism v. 4 software (unpaired *t-*test). Values represent the mean ± SD from at least three independent experiments.

We tested the phenotypic stability of the recoded virus variant HIV-PminA, HIV-GminA, HIV-GminB, HIV-GminC, and HIV-GminD and HIV-Pmax. We performed 15 serial virus passages in MT-4 cells, which corresponded to nearly 60 days of cell culture. Although virus deoptimized phenotypes were much stronger in primary PBMCs, serial passages were performed in MT-4 cells to maximize the number of viral replication cycles. Two replicate serial passages were started with a multiplicity of infection (MOI) of 0.0002. After 15 passages cultures were stopped and replication kinetic assays were performed for all variants and replicates. All deoptimized viruses except one replicate of HIV-GminA recovered the wild-type replication capacity in MT-4 cells (Figure 5). Optimized virus HIV-Pmax was still as fit as the wild-type virus in MT-4 cells. All viruses, including the wild-type virus HIV-Pmax, gained fitness after the passages in MT-4 cells (data not shown). These results demonstrate that deoptimized HIV-1 variants were able to recover fitness in tissue culture.

Clonal sequencing of the different deoptimized viral populations at passage 15 showed that several deoptimizing synonymous substitutions reverted to wild-type (Table 4). No significant reversions were observed in the optimized HIV-Pmax. The average mutation frequency in the deoptimized viral populations at passage 15 was between 1.5-fold and 12.0-fold higher than that of the wild-type population. There was a good correlation between the replication capacity value in MT-4 cells of the variant at passage 0 and the observed mutation frequency at passage 15: the lower the fitness the higher the mutation frequency (Table 4). The increase in mutation frequency between the mutant populations and wild-type population at passage 15 was statistically significant in all deoptimized viruses (*p* < 0.05; chi square test). In contrast, the optimized HIV-Pmax did not have significant increase in the population mutation frequency (*p* = 0.2425, chi square test). Reversion was observed in 3 out of the 15 mutations (20%) introduced in HIV-PminA. Similarly, 10 (8%), 15 (15%), 7 (8%) and 2 (6%) mutations reverted to wild-type on initially mutated positions in HIV-GminA, HIV-GminB, HIV-GminC and HIV-GminD, respectively (Table 4). In addition to the reversion of deoptimized synonymous substitutions, many additional synonymous and nonsynonymous mutations were observed (Table 4) (Additional file 4: Table S3 and Additional file 5: Table S4). Most of these additional mutations alleviate negative codon pair–bias scores (data not shown), strongly suggesting that phenotypic reversion was due to the reversion of some initially mutated residues together with the fixation of new mutations. Especially interesting was the high number of nonsynonymous substitutions found in some deoptimized gag populations. For instance, the HIV-GminA construct incorporated as majority variants the nonsynonymous mutations R4G, P48T and K114R in both passage replicates, indicating that these mutations were adaptive. The selection of these mutations or other mutations was not observed in the wild-type virus. The R4 and P48 gag positions are highly conserved in HIV-1. The R4G mutation was rarely observed in nature and the P48T mutation was not found in the alignment of 4644 gag sequences from Los Alamos database (http://www.hiv.lanl.gov/). Most of the nonsynonymous substitutions occurred in codons carrying deoptimized synonymous mutations, showing that the introduction of synonymous substitutions may allow the virus to explore an alternative nonsynonymous sequence space to improve viral fitness.

**Table 4:** Number of mutations and mutation frequency of recoded HIV-1 variants after 15 passages in MT-4 cells

	**Virus**	**Synonymous mutations (reversions)**^**a**^	**Nonsynonymous mutations**	**Mutations/Nucleotides**	**Mutation frequency**
Replicate 1	HIV-Pwtp15	3	9	12/6831	1.76 × 10^-3^
	HIV-Pmaxp15	2(2)	7	9/6237	1.44 × 10^-3^
	HIV-PminAp15	23(21)	9	32/7128	4.49 × 10^-3^
Replicate 2	HIV-Pwtp15	2	10	12/6237	1.92 × 10^-3^
	HIV-Pmaxp15	0	5	5/5049	0.99 × 10^-3^
	HIV-PminAp15	6(1)	12	18/5940	3.03 × 10^-3^
Replicate 1	HIV-GwtAp15	9	8	17/7875	2.16 × 10^-3^
	HIV-GminAp15	60(25)	59	119/6375	1.87 × 10^-2^
	HIV-GwtBp15	7	11	18/8421	2.14 × 10^-3^
	HIV-GminBp15	186(172)	5	191/7600	2.51 × 10^-2^
	HIV-GwtCp15	24	6	30/8400	3.57 × 10^-3^
	HIV-GminCp15	71(41)	26	97/8800	1.10 × 10^-2^
	HIV-GwtDp15	6	24	30/8190	3.66 × 10^-3^
	HIV-GminDp15	13(1)	15	28/4680	5.98 × 10^-3^
Replicate 2	HIV-GwtAp15	9	17	26/6375	4.08 × 10^-3^
	HIV-GminAp15	82(39)	78	160/8250	1.94 × 10^-2^
	HIV-GwtBp15	8	16	24/6000	4.00 × 10^-3^
	HIV-GminBp15	250(233)	8	250/9200	2.72 × 10^-2^
	HIV-GwtCp15	13	8	21/3600	5.83 × 10^-3^
	HIV-GminCp15	45(20)	19	64/4000	1.60 × 10^-2^
	HIV-GwtDp15	19	15	32/5460	5.86 × 10^-3^
	HIV-GminDp15	18(1)	24	42/4680	8.55 × 10^-3^

^a^Numbers in the parenthesis are reversions of mutated residues at passage 0.

## Discussion

Prior work has documented the effectiveness of making changes to the codon-pair bias of viral genomes in order to generate attenuated poliovirus and influenza virus [3,11]. We report here the application of this strategy for modifying the replicative capacity of HIV-1. The fact that very different viruses, such as poliovirus and influenza virus, are highly sensitive to codon-pair deoptimization suggests that this strategy may operate quite generally. However, no data were available regarding whether this approach would work with lentiviruses, which, in contrast to poliovirus and influenza virus, integrate into the host cell genome. In this study, we tested the extent to which codon-pair deoptimization and reoptimization of the HIV-1 genome could create viruses with altered replication capacity in cell culture.

We found that by codon pair–deoptimizing different moieties of the HIV-1 gag and pol genes it was possible to produce viruses with significantly lower viral replication capacity in MT-4 cells and PBMCs. These findings confirm the validity of using the SAVE strategy to attenuate lentiviruses ex vivo, in particular HIV-1. Our results provide also evidence that attenuated viruses may display decreased rates of protein translation of the targeted genes. After restricting viral replication to a single cycle by using a single-cycle HIV-1 vector, a significant reduction in protein production was observed in the vector carrying an attenuated virus variant. This reduction in protein synthesis was not accompanied by a reduction in the targeted RNA copy number, which suggests that translation, and not transcription, is implicated in the generation of the attenuated phenotype. Moreover, the lower RNA/p24 ratio found in these experiments for the deoptimized virus also suggests that there may be a defect in virus assembly. It has been hypothesized that the presence of a rare codon, marked by a synonymous polymorphism, affects the timing of protein folding and function [18]. This study therefore demonstrates that synonymous codon pair reengineering is an effective tool to phenotypically affect the replicative properties of HIV-1.

Codon-optimized HIV-1 genes significantly increased protein expression [6]. However, it was unknown whether HIV-1 codon reoptimization increases viral fitness or virulence. A high level of preferred human codon use runs the risk of disrupting some unknown property of the HIV-1 RNA that is deleterious for other reasons, e.g. disrupting the secondary structure of the HIV-1 RNA genome [19] or increasing the number of CpG islands [20]. Importantly, in our study, a protease optimized virus carrying 38 synonymous mutations was not attenuated and displayed a replication capacity similar to that of the wild-type virus in MT-4 cells and PBMCs. This result suggests that certain neutral genetic drift is operating in protease synonymous nucleotide residues. Thus, in addition to explore virus attenuation, synonymous codon pair recoding can be used to explore other aspects of the HIV-1 biology. Indeed, synonymous codon pair reoptimization has been used to overcome adeno-associated virus (AAV) Rep gene’s inhibitory effects on adenovirus replication [21], to identify unknown functional RNA elements in poliovirus coding sequences [22] and to study poliovirus evolvability and pathogenesis [23].

Previously, it was found that a codon pair base deoptimized poliovirus variant containing 224 synonymous substitutions did not revert its phenotype after 19 passages in HeLaR19 cells [3]. In contrast, we show here that highly attenuated HIV-1 variants carrying more or near one hundred synonymous substitutions reverted their phenotype after 15 passages in MT4-4 cells. Most notably, sequence clonal analysis of phenotypically reverted viral populations shows that phenotype reversion is due not only to the reversion of initially introduced synonymous mutations but also to the presence of new synonymous and nonsynonymous mutations. Of note, no genetic or phenotypic reversion is observed in a codon pair base optimized viral population after the same number of passages in MT-4 cells. Differences in the life cycle between poliovirus and HIV-1 may account for the above discrepancies. Alternatively, differences in virus protein or genome mutational robustness and evolvability can not be discarded. The different codon usage between poliovirus and HIV-1 may determine differences in their mutational robustness and evolutionary capacity [23,24]. A higher number of substitutions may better prevent reversion to the wild-type genotype. Instead, other HIV-1 genomic regions can be explored to determine whether other structural or accessory genes can be targeted with SAVE to generate recoded virus with a higher genetic barrier for phenotype reversion.

This study has some limitations that are worth noting. First, despite the fact that reduction in protein synthesis was not accompanied by a reduction in the targeted HIV-1 RNA copy number, RNA sequences may be also targeted by factors other than those of the translation machinery (e.g. aberrant splicing, RNA decay process or miRNA targeting). Second, although we obtained a robust attenuation in PBMCs from healthy donors, our variants should be tested in an animal model, ideally the SIV macaque model, in order to demonstrate in vivo attenuation. Animal experiments should include optimized viruses to test whether these optimized variants are also not attenuated in vivo. Proof that lower replication capacities ex vivo can be translated into in vivo attenuation come from studies of the evolution of resistance to antiretroviral drugs, which is characterized by significant costs in ex vivo replication capacity [25]. Future work should include the immunization of animal models with different codon pair–deoptimized attenuated HIV-1 strains to investigate whether recoded viruses are also attenuated in vivo and to determine their long-term stability.

## Conclusions

Our results demonstrate that codon pair–deoptimization of different moieties of the HIV-1 gag and pol genes produce viruses with significantly lower viral replication capacity in MT-4 cells and PBMCs. This study therefore demonstrates that synonymous codon pair reengineering is an effective tool to phenotypically affect the replicative properties of lentiviruses, in particular HIV-1.

## Methods

### Cell lines

MT-4 cells were obtained from the National Institutes of Health (NIH) AIDS Research and Reference Reagent Program. HEK 293 T cells were obtained from the American Type Culture Collection (ATCC). MT-4 cells were grown in Roswell Park Memorial Institute (RPMI) 1640 L-glutamine medium supplemented with 10% heat-inactivated fetal bovine serum (FBS) (Gibco). The 293 T cells were grown in Dulbecco’s Modified Eagle Medium (DMEM) (Gibco) supplemented with 10% heat-inactivated FBS. For isolation of PBMC blood was taken from healthy donors. PBMCs were provided by the Banc de Sang i de Teixits (BST, Barcelona, Spain) after approval from the Germans Trias i Pujol hospital ethics committee. PBMCs purified by ficoll density gradient were stimulated with phytohemagglutinin (PHA) and interleukin-2 (IL2) (see below) and maintained in RPMI medium supplemented with 20% FBS.

### Generation of synthetic HIV-1

All of the synthetic HIV-1 variants used in this study are based on the HXB2 strain (http://www.hiv.lanl.gov). Synthetic HIV-1 protease was generated by combining three overlapping synthetic DNA oligonucleotides (Integrated DNA Technologies) by PCR; we used the overlap extension protocol described in our previous paper [26]. PCRs were purified with the QIAquick PCR Purification Kit (QIAGEN) and sequenced to confirm the desired sequence. Sequencing was performed with the Big Dye v3.1 kit and the 3100 DNA sequencing system (Applied Biosystems). Similarly, synthetic HIV-1 gag was generated by combining eight overlapping synthetic DNA oligonucleotides. Sequencing oligonuclotides for protease and gag were described previously [27].

Codon pair score is defined as the natural log of the ratio of the observed over the expected number of occurrences of each codon pair over all human coding regions [3]. The expected frequency is calculated based on the relative proportion of the number of times an amino acid is encoded by a specific codon. A positive codon pair score value signifies that the given codon pair is statistically over-represented, and a negative codon pair score indicates the pair is statistically under-represented in the human genome. Using these calculated codon pair scores, the codon pair bias for an entire open reading frame can then be calculated as the arithmetic mean of the individual codon pair scores (Tables 1 and 3).

Recombinant infectious viruses were generated as we described previously [27,28]. Briefly, 100 ng of full-length protease or 500 ng of gag purified PCR products were cotransfected into MT-4 cells with 1 μg of protease-deleted [29] or gag-protease-deleted [30] HXB2 clone that had been previously linearized with BstE II (New England Biolabs). Cell culture supernatants were harvested on days 3, 5, and 7 after transfection when the concentration of HIV-1 p24 antigen (Genscreen HIV-1 Ag assay, Bio-Rad) surpassed 500 ng/ml. If p24 antigen was not detected after 7 days of culture, five blind passages, feeding the cultures with fresh medium and new MT-4 cells, were performed to recover viable virus. After five blind passages without p24 antigen detection in the cultured supernatant, the construct was considered nonviable in MT-4 cell culture. Virus titration was performed in MT-4 cells, and values were expressed as tissue culture dose for 50% infectivity (TCID_50_), as previously described [31].

To control the folding free energy of the RNA of the recoded constructs, we determined their RNA folding energies with the program mfold [32], as previously described [3]. mfold prevents the creation of any regions with large secondary structures, such as stable hairpins or stem loops. To ensure that changes in synonymous codons do not systematically reduce codon bias, the effective number of codons (ENC) was also calculated by use of the Codon W program (http://mobyle.pasteur.fr) [33], as previously described [34].

### Replication capacity assays

Viral replication kinetics were performed by infecting either 1 × 10^6^ MT-4 cells with 200 TCID_50_ (MOI of 0.0002) or 2 × 10^6^ PBMCs (mixed from four healthy donors), stimulated 3 days previously with 5 μg/ml PHA (Sigma-Aldrich) and 10 U/ml IL2, with 2000 TCID_50_ of each viral stock (MOI 0.001). After 4 h of incubation at 37°C and 5% CO_2_, cells were washed twice with phosphate-buffered saline (PBS) and resuspended in RPMI medium supplemented with 10% FBS (MT-4 cells) or 20% FBS plus 10 U/ml IL2 (PMBCs). Every 24 h, 200 μl of supernatant was removed for p24 antigen quantification. Viral replication was quantified by measuring HIV-1 capsid p24 antigen production in the culture supernatant for 5 days (MT-4) or 7 days (PBMCs). Growth kinetics were analyzed by fitting a linear model to the log-transformed p24 data during the exponential growth phase by maximum likelihood methods as we described previously [35].

### Growth competition assays

Dual competition experiments were performed by infecting 1 × 10^6^ MT-4 cells with each pair of competing viruses mixed at different ratios with an overall TCID_50_ of 1000 (MOI of 0.001). HIV-PminB and HIV-PminC were independently competed with the HXB2 wild-type virus. After 4 h at 37°C, cells were washed twice with PBS, resuspended in 5 ml of medium, and cultured in six-well plates. Culture supernatants were collected twice weekly, and 100 μl of supernatant was used to infect fresh cells. Viral RNA was extracted and amplified by one-step RT-PCR with oligonucleotides 5′prot 2 and 3′prot 2. Ratios of the two competing variants were estimated on days 0, 7, 14, and 21, based on the relative peak heights of all the different mutations in electropherograms obtained by bidirectional sequencing of the protease coding region.

### Single-cycle infectivity assay

Recoded HIV-1 proteases and wild-type HXB2 protease were cloned into the GFP reporter HIV-1 infectious clone pNL4-3-deltaE-EGFP (NIH AIDS Research and Reference Reagent Program). Protease sequences were PCR amplified with oligonuclotides Apa1988 (5′-ACATAGCCAAAAATTGCAGGGCCCCTAG-3′) and Sbf2838 (5′-CTGATTTTTTCTGTTTTAACCCTGCAGGATG-3′), digested with Apa I and Sbf I (New England Biolabs), and cloned into pNL4-3-deltaE-EGFP between the Apa I and Sbf I sites. Recombinant pNL4-3-deltaE-EGFP plasmids (4 μg) were used to transfect 8 × 10^5^ 293 T cells in the presence of Lipofectamine 2000 (Invitrogen). The relative replication capacity of the virus was determined by measuring the amount of p24 antigen produced 72 h after transfection. Replication capacity is expressed as the percentage of p24 antigen produced by the vectors containing each of the derived protease sequences compared to the p24 antigen from the vector containing the HIV-1 HXB2 protease reference sequence (100%). Replication capacity measurements were normalized for differences in transfection efficiencies by monitoring the GFP activity (by flow cytometry) generated in transfected cells.

### Real-time quantitative RT-PCR analysis of HIV-1 gag (p24) and GFP genes

Real-time quantitative RT-PCR analysis was used to quantify HIV-1 gag RNA. A total of 8 × 10^5^ 293 T cells were transfected with 4 μg of recombinant pNL4-3-deltaE-EGFP plasmid. Total cell RNA was isolated (High Pure RNA Isolation Kit, Roche) 72 h post-transfection. Reverse transcription was performed with the SuperScript III First-Strand Synthesis System for RT-PCR (Invitrogen) in a 20 μl reaction volume containing 1 μg of total RNA. Real-time quantitative RT-PCR analysis of gag (p24) was carried out with TaqMan Universal Master Mix (Applied Biosystems) and the oligonucleotides gag (p24) Fw (5′-AAGGCCAGGGAATTTTCTTC −3′) and gag (p24) Rv (5′-TATTGTGACGAGGGGTCGTT −3′) and the probe FAM (5′-ACTGGCAGAAAACAGGGAGA-3′) TAMRA. In addition to the real-time quantitative RT-PCR, analysis of GFP was performed by using the primers rightGFP (5′-CCATGCCGAGAGTGATCC-3′) and leftGFP (5′-CGACAACCACTACCTGAGCA-3′) and the probe FAM (5′-ACATGGTCCTGCTGGAGTTC-3′) TAMRA. The endogenous gene GAPDH (Applied Biosystems) was used as a control. Relative quantification of both mRNAs was calculated as described elsewhere [36].

### HIV-1 drug susceptibility tests

Reverse transcriptase and protease inhibitors were obtained from the NIH AIDS Research and Reference Reagent Program. After virus propagation and titration, HIV-1 drug susceptibility data were obtained in MT-4 cells as previously described [35].

### Serial passage experiments

We tested the phenotypic stability of attenuated viruses by serially propagating the HIV-1 recoded variants in MT-4 cells. Briefly, wild-type virus, HIV-PminA, HIV-Pmax, HIV-GminA, HIV-GminB, HIV-GminC and HIV-GminD were added at a MOI of 0.0002 to 1 × 10^6^ MT-4 cells. MT-4 cells were maintained in RPMI medium supplemented with 10% FBS. After 4 days, one-tenth of the culture, including cells and supernatant, was transferred to 1 × 10^6^ fresh MT-4 cells. All virus passages were performed in duplicate. Virus production was monitored by measuring p24 antigen.

Viral genomic RNA from 140 μl of culture supernatant was purified with the QIAamp Viral RNA Kit (QIAGEN). After the viral RNA was isolated, 5 μl of resuspended RNA was reverse transcribed and PCR amplified with the SuperScript III First-Strand Synthesis System for RT-PCR (Invitrogen) and 10 pmol of the corresponding gag or protease oligonucleotides as we described previously [27,37]. The amplified RNA was cloned into the pGEM-T Easy Vector System I (Promega). The DNA inserts were sequenced by using the flanking oligonucleotides sp6, T7 and internal gag oligonucleotides.

### Statistical analysis

Unpaired *t*-test and chi square test were performed using GraphPad Prism version 4.00 for Windows.

### Nucleotide sequence accession numbers

The sequences reported in this paper, including synthetically recoded HIV-1 variants, have been deposited in the GenBank database [accession numbers JX961631(HIV-Pmin, JX961632 (HIV-Pmax) and JX961633 (HIV-Gmin)].

## Competing interests

The authors have declared that no competing interests exist.

## Authors’ contributions

GM carried out the generation of synthetic HIV-1 constructs, the virus replication capacity kinetics, the single-cycle infectivity assay, participated in the design of the study and drafted the manuscript. MN carried out real-time quantitative RT-PCR analysis of HIV-1 p24 and GFP genes and the serial passage experiments. CA carried out the HIV-1 drug susceptibility tests and virus quasispecies cloning and sequencing, BC participated in data analysis and statistical analysis. MAM conceived and designed the study and wrote the manuscript. All authors read and approved the final manuscript.

## Supplementary Material

Additional file 1: Table S1 ENC^a^ and GC_3_S^b^ values for recoded HIV-1 constructs. Click here for file

Additional file 2: Figure S1 Folding energy of recoded HIV-1 constructs. To ensure that strong secondary structures do not affect translation efficiency, we scanned the (A) protease and (B) gag region of our constructs using the program mFold (see Methods). The analysis was performed on 100-base long segments, having 80 bases overlap with each other. Segments with lower binding energy than a threshold of -30Kcal/mol will incur random synonymous substitutions at C-G binding locations, such that the binding energy of the segment could be elevated. Click here for file

Additional file 3: Table S2 Susceptibility of recoded HIV-1 variants to reverse transcriptase and protease inhibitors. Click here for file

Additional file 4: Table S3 Synonymous mutations of recoded HIV-1 variants after 15 passages in MT-4 cells. Click here for file

Additional file 5: Table S4 Nonsynonymous mutations of recoded HIV-1 variants after 15 passages in MT-4 cells. Click here for file
