# Supplementary material for: Changes in codon-pair bias of human immunodeficiency virus type 1 have profound effects on virus replication in cell culture
Source: Retrovirology. 2013 Jul 25;10:78. doi: 10.1186/1742-4690-10-78 (PMC3726367; doi:10.1186/1742-4690-10-78)
Supplement: Additional file 1: Table S1 — ENCa and GC3Sb values for recoded HIV-1 constructs. [file 1742-4690-10-78-S1.doc]

**Additional file 1: Table S1.** ENC^a^ and GC_3_S^b^ values for recoded HIV-1 constructs.

| HIV-1 constructs | ENC | GC_3_S |
| --- | --- | --- |
| HIV-Pwt | 40.290 | 0.232 |
| HIV-Pmax | 39.780 | 0.200 |
| HIV-PminA | 39.640 | 0.295 |
| HIV-PminB | 52.320 | 0.337 |
| HIV-PminC | 54.240 | 0.326 |
| HIV-PminAB | 57.170 | 0.421 |
| HIV-PminAC | 53.060 | 0.379 |
| HIV-PminBC | 53.160 | 0.453 |
| HIV-Pmin | 54.930 | 0.537 |
| HIV-Gwt | 45.330 | 0.352 |
| HIV-GminA | 50.560 | 0.400 |
| HIV-GminB | 49.810 | 0.388 |
| HIV-GminC | 52.280 | 0.410 |
| HIV-GminD | 47.940 | 0.360 |
| HIV-GminAB | 52.270 | 0.435 |
| HIV-GminAC | 54.680 | 0.458 |
| HIV-GminAD | 52.530 | 0.408 |
| HIV-GminBC | 53.950 | 0.446 |
| HIV-GminBD | 53.030 | 0.396 |
| HIV-GminCD | 53.260 | 0.419 |
| HIV-GminABC | 52.440 | 0.494 |
| HIV-GminABD | 54.150 | 0.444 |

^a^ENC. effective number of codons. ^b^GC_3_S. the frequency of use of G+C in synonymous variable third positions of codons.
