# Supplementary material for: Changes in codon-pair bias of human immunodeficiency virus type 1 have profound effects on virus replication in cell culture
Source: Retrovirology. 2013 Jul 25;10:78. doi: 10.1186/1742-4690-10-78 (PMC3726367; doi:10.1186/1742-4690-10-78)
Supplement: Additional file 3: Table S2 — Susceptibility of recoded HIV-1 variants to reverse transcriptase and protease inhibitors. [file 1742-4690-10-78-S3.doc]

**Additional file 3: Table S2.** Susceptibility of recoded HIV-1 variants to reverse transcriptase and protease inhibitors.

| IC50 (nM) | | | | | | |
| --- | --- | --- | --- | --- | --- | --- |
|  | AZT | TNF-DF | NFV | DRV | ATV | APV |
| HIV-Pwt | 15,33 ± 3,61 | 27,32 ± 5,41 | 11,44 ± 4,68 | 1,69 ± 0,31 | 3,19 ± 0,74 | 14,43 ± 1,29 |
| HIV-Pmax | 29,02 ± 3,65 (1,89) | 36,83 ± 7,31 (1,35) | 12,63 ± 3,31 (1,1) | 2,93 ± 0,07 (1,73) | 3,57 ± 1  (1,12) | 15,11 ± 2,72 (1,05) |
| HIV-PminA | 18,25 ± 9,5 (1,19) | 34,18 ± 1,94 (1,25) | 13,73 ± 7,23 (1,2) | 2,12 ± 0,96 (1,25) | 3,57 ± 0,53 (1,12) | 18,47 ± 1,69 (1,28) |
| HIV-PminB | 14,07 ± 8,10 (0,92) | 33,98 ± 3,36 (1,24) | 12,01 ± 1,80 (1,05) | 2,83 ± 0,20 (1,67) | 3,02 ± 0,74 (0,95) | 17,68 ± 2,03 (1,22) |
| HIV-PminC | 16,08 ± 6,94 (1,05) | 34,28 ± 4,76 (1,25) | 12,03 ± 4,32 (1,05) | 3,03 ± 0,51 (1,79) | 3,62 ± 0,65 (1,13) | 17,74 ± 2,29 (1,23) |
| HIV-PminBC | 14,24 ± 5,02 (0,93) | 28,81 ± 4,20 (1,05) | 9,84 ± 2,71 (0,86) | 2,77 ± 0,25 (1,64) | 4,41 ± 0,49 (1,38) | 16,38 ± 2,91 (1,13) |
|  |  |  |  |  |  |  |
| HIV-Gwt | 6,06 ± 1,14 | 18,30 ± 3,41 | 9,93 ± 3,04 | 2,37 ± 0,71 | 4,44 ± 1,28 | 13,56 ± 1,98 |
| HIV-GminA | 6,68 ± 2,12 (1,10) | 13,64 ± 8,85 (0,75) | 3,39 ± 1,26 (0,34) | 1,74 ± 1,24 (0,73) | 2,08 ± 0,96 (0,47) | 8,11 ± 4,96 (0,60) |
| HIV-GminB | 12,19 ± 1,96 (2,01) | 28,73 ± 8,86 (1,57) | 10,14 ± 8,03 (1,02) | 2,59 ± 0,69 (1,09) | 4,25 ± 0,82 (0,96) | 14,66 ± 1,63 (1,08) |
| HIV-GminD | 8,64 ± 3,30 (1,42) | 27,31 ± 9,23 (1,5) | 8,66 ± 4,36 (0,87) | 2,20 ± 0,14 (0,93) | 3,13 ± 1,54 (0,7) | 12,63 ± 2,04 (0,93) |

The IC50 values represent averages ± standard deviations of at least three tests. The fold increase in IC50 relative to the wild type HXB2 virus control is shown between parentheses.
