# Supplementary material for: Changes in codon-pair bias of human immunodeficiency virus type 1 have profound effects on virus replication in cell culture
Source: Retrovirology. 2013 Jul 25;10:78. doi: 10.1186/1742-4690-10-78 (PMC3726367; doi:10.1186/1742-4690-10-78)
Supplement: Additional file 4: Table S3 — Synonymous mutations of recoded HIV-1 variants after 15 passages in MT-4 cells. [file 1742-4690-10-78-S4.doc]

**Additional file 4: Table S3.** Synonymous mutations of recoded HIV-1 variants after 15 passages in MT-4 cells.

|  | Virus | Nucleotide position^a^ | Starting nucleotide | Reversion to wt virus | Other mutation | Number of clones |
| --- | --- | --- | --- | --- | --- | --- |
| Replicate 1 | HIV-Pwtp15 | 207 | T |  | C | 1/24 |
|  |  | 210 | A |  | G | 1/24 |
|  |  | 261 | A |  | G | 1/24 |
|  | HIV-Pmaxp15 | 81 | G | A |  | 1/21 |
|  |  | 295 | T | C |  | 1/21 |
|  | HIV-PminAp15 | 9 | C |  | T | 1/24 |
|  |  | 72 | C |  | T | 1/24 |
|  |  | 97 | C | T |  | 10/24 |
|  |  | 99 | C | A |  | 10/24 |
| Replicate 2 | HIV-Pwtp15 | 66 | T |  | C | 1/21 |
|  |  | 285 | C |  | T | 1/21 |
|  | HIV-Pmaxp15 | - | - | - | - | - |
|  | HIV-PminAp15 | 30 | C |  | T | 1/20 |
|  |  | 57 | G | A |  | 1/20 |
|  |  | 78 | C |  | T | 1/20 |
|  |  | 93 | C |  | T | 1/20 |
|  |  | 195 | A |  | G | 1/20 |
|  |  | 290 | T |  | A | 1/20 |
| Replicate 1 | HIV-GwtAp15 | 87 | T |  | C | 1/21 |
|  |  | 96 | A |  | G | 1/21 |
|  |  | 174 | A |  | G | 1/21 |
|  |  | 201 | C |  | T | 1/21 |
|  |  | 246 | A |  | G | 1/21 |
|  |  | 264 | A |  | G | 1/21 |
|  |  | 279 | G |  | A | 1/21 |
|  |  | 336 | G |  | A | 1/21 |
|  |  | 345 | A |  | G | 1/21 |
|  | HIV-GminAp15 | 18 | C |  | T | 5/17 |
|  |  | 42 | T |  | C | 10/17 |
|  |  | 60 | C |  | A | 1/17 |
|  |  | 66 | C | G |  | 9/17 |
|  |  | 162 | C |  | T | 14/17 |
|  |  | 165 | G | A |  | 1/17 |
|  |  | 189 | A | G |  | 2/17 |
|  |  | 201 | T | C |  | 8/17 |
|  |  | 216 | C |  | T | 1/17 |
|  |  | 258 | C | T |  | 4/17 |
|  |  | 291 | T | C |  | 1/17 |
|  |  | 303 | C | A |  | 1/17 |
|  |  | 333 | G |  | A | 3/17 |
|  | HIV-GwtBp15 | 417 | G |  | A | 1/21 |
|  |  | 489 | T |  | C | 1/21 |
|  |  | 504 | G |  | A | 1/21 |
|  |  | 621 | G |  | A | 1/21 |
|  |  | 705 | C |  | T | 1/21 |
|  |  | 720 | T |  | C | 1/21 |
|  |  | 755 | A |  | C | 1/21 |
|  | HIV-GminBp15 | 369 | A |  | C | 1/19 |
|  |  | 378 | C | T |  | 18/19 |
|  |  | 384 | A | C |  | 18/19 |
|  |  | 385 | T | A |  | 18/19 |
|  |  | 386 | C | G |  | 18/19 |
|  |  | 387 | G | C |  | 18/19 |
|  |  | 399 | G | T |  | 18/19 |
|  |  | 399 | G |  | A | 1/19 |
|  |  | 408 | A | G |  | 18/19 |
|  |  | 411 | T | C |  | 18/19 |
|  |  | 429 | G | A |  | 14/19 |
|  |  | 435 | A | G |  | 7/19 |
|  |  | 477 | C |  | T | 1/19 |
|  |  | 612 | G | C |  | 2/19 |
|  |  | 612 | G |  | A | 3/19 |
|  |  | 615 | T | C |  | 1/19 |
|  |  | 687 | C | A |  | 1/19 |
|  |  | 711 | C | A |  | 3/19 |
|  |  | 714 | A |  | G | 1/19 |
|  |  | 717 | T |  | C | 7/19 |
|  | HIV-GwtCp15 | 762 | A |  | G | 1/21 |
|  |  | 870 | G |  | A | 21/21 |
|  |  | 903 | T |  | C | 1/21 |
|  |  | 927 | T |  | C | 1/21 |
|  | HIV-GminCp15 | 822 | T |  | G | 1/22 |
|  |  | 843 | G |  | A | 1/22 |
|  |  | 906 | G | A |  | 1/22 |
|  |  | 921 | A | G |  | 1/22 |
|  |  | 978 | T |  | C | 22/22 |
|  |  | 984 | G | A |  | 22/22 |
|  |  | 1008 | G | A |  | 1/22 |
|  |  | 1044 | G | A |  | 15/22 |
|  |  | 1110 | A |  | G | 6/22 |
|  |  | 1113 | G | A |  | 1/22 |
|  | HIV-GwtDp15 | 1146 | T |  | C | 1/21 |
|  |  | 1151 | G |  | A | 1/21 |
|  |  | 1335 | A |  | G | 1/21 |
|  |  | 1380 | A |  | G | 1/21 |
|  |  | 1449 | G |  | A | 1/21 |
|  | HIV-GminDp15 | 1121 | C |  | T | 1/12 |
|  |  | 1170 | T |  | A | 1/12 |
|  |  | 1215 | C |  | T | 1/12 |
|  |  | 1221 | G |  | A | 1/12 |
|  |  | 1224 | A |  | C | 1/12 |
|  |  | 1248 | C | T |  | 1/12 |
|  |  | 1386 | C |  | T | 4/12 |
|  |  | 1395 | T |  | C | 3/12 |
| Replicate 2 | HIV-GwtAp15 | 18 | A |  | G | 1/17 |
|  |  | 144 | T |  | G | 2/17 |
|  |  | 174 | A |  | G | 1/17 |
|  |  | 195 | A |  | G | 3/17 |
|  |  | 294 | G |  | A | 1/17 |
|  |  | 318 | A |  | G | 1/17 |
|  | HIV-GminAp15 | 18 | C |  | T | 1/22 |
|  |  | 42 | T |  | C | 16/22 |
|  |  | 51 | G | A |  | 1/22 |
|  |  | 66 | C | G |  | 14/22 |
|  |  | 69 | C |  | T | 1/22 |
|  |  | 90 | G | A |  | 3/22 |
|  |  | 123 | C |  | G | 1/22 |
|  |  | 162 | C |  | T | 20/22 |
|  |  | 201 | T | C |  | 15/22 |
|  |  | 258 | C | T |  | 1/22 |
|  |  | 291 | C | T |  | 2/22 |
|  |  | 306 | T | C |  | 3/22 |
|  |  | 333 | G |  | A | 4/22 |
|  | HIV-GwtBp15 | 393 | T |  | C | 1/15 |
|  |  | 405 | G |  | A | 1/15 |
|  |  | 420 | G |  | A | 1/15 |
|  |  | 453 | T |  | C | 1/15 |
|  |  | 477 | G |  | A | 1/15 |
|  |  | 495 | C |  | T | 1/15 |
|  |  | 675 | A |  | G | 1/15 |
|  |  | 711 | A |  | G | 1/15 |
|  | HIV-GminBp15 | 378 | C | T |  | 23/23 |
|  |  | 384 | A | C |  | 23/23 |
|  |  | 385 | T | A |  | 23/23 |
|  |  | 386 | C | G |  | 23/23 |
|  |  | 387 | G | C |  | 23/23 |
|  |  | 399 | G | T |  | 23/23 |
|  |  | 408 | A | G |  | 23/23 |
|  |  | 411 | T | C |  | 23/23 |
|  |  | 429 | G | A |  | 23/23 |
|  |  | 435 | A | G |  | 14/23 |
|  |  | 471 | G | A |  | 1/23 |
|  |  | 477 | C |  | T | 1/23 |
|  |  | 510 | G |  | A | 2/23 |
|  |  | 534 | A |  | G | 2/23 |
|  |  | 558 | G |  | A | 3/23 |
|  |  | 612 | G | C |  | 2/23 |
|  |  | 612 | G |  | A | 5/23 |
|  |  | 615 | T | C |  | 3/23 |
|  |  | 639 | T |  | C | 1/23 |
|  |  | 687 | C | A |  | 6/23 |
|  |  | 711 | C | T |  | 1/23 |
|  |  | 717 | T |  | C | 3/23 |
|  | HIV-GwtCp15 | 822 | A |  | G | 1/9 |
|  |  | 870 | G |  | A | 9/9 |
|  |  | 915 | A |  | G | 1/9 |
|  |  | 918 | C |  | T | 1/9 |
|  | HIV-GminCp15 | 876 | G | C |  | 1/10 |
|  |  | 895 | C |  | A | 1/10 |
|  |  | 906 | G | A |  | 1/10 |
|  |  | 978 | T |  | C | 10/10 |
|  |  | 984 | G | A |  | 10/10 |
|  |  | 1044 | G | A |  | 8/10 |
|  |  | 1110 | A |  | G | 2/10 |
|  | HIV-GwtDp15 | 1176 | T |  | C | 1/14 |
|  |  | 1197 | G |  | A | 1/14 |
|  |  | 1405 | A |  | G | 3/14 |
|  |  | 1470 | G |  | A | 14/14 |
|  | HIV-GminDp15 | 1146 | C | T |  | 1/12 |
|  |  | 1154 | A |  | G | 1/12 |
|  |  | 1188 | A |  | T | 1/12 |
|  |  | 1203 | C |  | T | 1/12 |
|  |  | 1221 | G |  | A | 1/12 |
|  |  | 1413 | T |  | C | 1/12 |
|  |  | 1470 | G |  | A | 1/12 |

^a^protease and gag nucleotide numbering
