# Supplementary material for: Changes in codon-pair bias of human immunodeficiency virus type 1 have profound effects on virus replication in cell culture
Source: Retrovirology. 2013 Jul 25;10:78. doi: 10.1186/1742-4690-10-78 (PMC3726367; doi:10.1186/1742-4690-10-78)
Supplement: Additional file 5: Table S4 — Nonsynonymous mutations of recoded HIV-1 variants after 15 passages in MT-4 cells. [file 1742-4690-10-78-S5.doc]

**Additional file 5: Table S4.** Nonsynonymous mutations of recoded HIV-1 variants after 15 passages in MT-4 cells.

|  | Virus | Mutation^a^ | Number of clones |
| --- | --- | --- | --- |
| Replicate 1 | HIV-Pwtp15 | D25G | 1/24 |
|  |  | M46L | 1/24 |
|  |  | G49E | 1/24 |
|  |  | V56I | 1/24 |
|  |  | P79L | 1/24 |
|  |  | V82I | 1/24 |
|  |  | G94D | 1/24 |
|  |  | N98P | 1/24 |
|  | HIV-Pmaxp15 | G27R | 1/21 |
|  |  | G27E | 1/21 |
|  |  | S37P | 4/21 |
|  |  | L90S | 1/21 |
|  | HIV-PminAp15 | G17R | 1/24 |
|  |  | G27R | 1/24 |
|  |  | M36I | 1/24 |
|  |  | G40E | 1/24 |
|  |  | R41K | 1/24 |
|  |  | G48R | 1/24 |
|  |  | K55Q | 1/24 |
|  |  | Y59D | 1/24 |
|  |  | C95Y | 1/24 |
| Replicate 2 | HIV-Pwtp15 | L23V | 1/21 |
|  |  | G27R | 1/21 |
|  |  | I54M | 1/21 |
|  |  | D60N | 1/21 |
|  |  | L63V | 2/21 |
|  |  | E65K | 1/21 |
|  |  | K70I | 1/21 |
|  |  | Q92R | 1/21 |
|  | HIV-Pmaxp15 | T26I | 1/17 |
|  |  | L33F | 1/17 |
|  |  | S37P | 3/17 |
|  | HIV-PminAp15 | D29N | 1/20 |
|  |  | R41K | 1/20 |
|  |  | W42STOP | 1/20 |
|  |  | M46I | 1/20 |
|  |  | G49E | 1/20 |
|  |  | G49R | 1/20 |
|  |  | V56I | 1/20 |
|  |  | Q61E | 2/20 |
|  |  | L63V | 1/20 |
|  |  | E65K | 1/20 |
|  |  | G68R | 1/20 |
| Replicate 1 | HIV-GwtAp15 | G11R | 1/21 |
|  |  | G11E | 1/21 |
|  |  | K18R | 2/21 |
|  |  | G49D | 1/21 |
|  |  | C87Y | 1/21 |
|  |  | K110E | 1/21 |
|  |  | A119T | 1/21 |
|  | HIV-GminAp15 | R4G | 6/17 |
|  |  | R15Q | 1/17 |
|  |  | E40K | 1/17 |
|  |  | V46I | 3/17 |
|  |  | P48T | 14/17 |
|  |  | G49E | 1/17 |
|  |  | I60V | 5/17 |
|  |  | V82I | 1/17 |
|  |  | T84A | 7/17 |
|  |  | E105K | 2/17 |
|  |  | K114R | 17/17 |
|  |  | A115V | 1/17 |
|  | HIV-GwtBp15 | N126Y | 1/21 |
|  |  | N126S | 1/21 |
|  |  | A146T | 1/21 |
|  |  | Q199R | 1/21 |
|  |  | V215A | 6/21 |
|  |  | T251A | 1/21 |
|  | HIV-GminBp15 | V164I | 1/19 |
|  |  | V173I | 4/19 |
|  | HIV-GwtCp15 | T251A | 1/21 |
|  |  | N255T | 1/21 |
|  |  | S281N | 1/21 |
|  |  | Q287N | 1/21 |
|  |  | T320A | 1/21 |
|  |  | A341V | 1/21 |
|  | HIV-GminCp15 | R286C | 1/22 |
|  |  | T320A | 1/22 |
|  |  | S368R | 1/22 |
|  |  | V370A | 16/22 |
|  |  | T371M | 7/22 |
|  | HIV-GwtDp15 | F383I | 1/21 |
|  |  | R384K | 15/21 |
|  |  | V390G | 1/21 |
|  |  | T401I | 1/21 |
|  |  | R409K | 1/21 |
|  |  | M423L | 1/21 |
|  |  | S451N | 1/21 |
|  |  | P453T | 1/21 |
|  |  | T469A | 1/21 |
|  | HIV-GminDp15 | M377T | 1/12 |
|  |  | F283S | 1/12 |
|  |  | N385S | 1/12 |
|  |  | V390I | 1/12 |
|  |  | K391R | 1/12 |
|  |  | T401I | 1/12 |
|  |  | N404S | 2/12 |
|  |  | R406G | 6/12 |
|  |  | A407V | 1/12 |
| Replicate 2 | HIV-GwtAp15 | E17Q | 1/17 |
|  |  | K18R | 2/17 |
|  |  | P23A | 2/17 |
|  |  | W36G | 2/17 |
|  |  | L50R | 2/17 |
|  |  | L61R | 2/17 |
|  |  | C87W | 2/17 |
|  |  | V88G | 2/17 |
|  |  | K112R | 1/17 |
|  | HIV-GminAp15 | A3T | 1/22 |
|  |  | R4G | 15/22 |
|  |  | K30R | 1/22 |
|  |  | V35I | 1/22 |
|  |  | E40G | 1/22 |
|  |  | V46I | 2/22 |
|  |  | P48T | 20/22 |
|  |  | I60V | 1/22 |
|  |  | T84A | 5/22 |
|  |  | E105K | 2/22 |
|  |  | N109S | 1/22 |
|  |  | K110R | 1/22 |
|  |  | K113T | 1/22 |
|  |  | K113N | 1/22 |
|  |  | K114R | 22/22 |
|  |  | A115V | 1/22 |
|  |  | Q117L | 1/22 |
|  |  | A119V | 1/22 |
|  | HIV-GwtBp15 | N126S | 1/15 |
|  |  | V128G | 2/15 |
|  |  | V135G | 2/15 |
|  |  | M142R | 2/15 |
|  |  | N153K | 2/15 |
|  |  | W155Q | 2/15 |
|  |  | Q199A | 1/15 |
|  |  | V215A | 3/15 |
|  | HIV-GminBp15 | V128I | 1/23 |
|  |  | I138T | 1/23 |
|  |  | A174T | 1/23 |
|  |  | V191I | 4/23 |
|  |  | W212STOP | 1/23 |
|  | HIV-GwtCp15 | A341S | 1/9 |
|  |  | A341V | 1/9 |
|  |  | G357S | 2/9 |
|  |  | G357R | 1/9 |
|  |  | S373T | 1/9 |
|  | HIV-GminCp15 | K290E | 1/10 |
|  |  | F293V | 1/10 |
|  |  | V297L | 1/10 |
|  |  | Y301STOP | 1/10 |
|  |  | A340V | 1/10 |
|  |  | V370A | 8/10 |
|  |  | T371M | 5/10 |
|  |  | A374T | 1/10 |
|  | HIV-GwtDp15 | S374T | 1/14 |
|  |  | T376I | 1/14 |
|  |  | R386K | 1/14 |
|  |  | V391G | 1/14 |
|  |  | V391G | 4/14 |
|  |  | H422P | 1/14 |
|  |  | M424L | 2/14 |
|  |  | G435STOP | 1/14 |
|  |  | L450V | 1/14 |
|  |  | E455G | 1/14 |
|  |  | T502A | 1/14 |
|  | HIV-GminDp15 | N382F | 1/12 |
|  |  | N385S | 1/12 |
|  |  | I389V | 1/12 |
|  |  | V390I | 2/12 |
|  |  | E398G | 1/12 |
|  |  | N404S | 3/12 |
|  |  | R406G | 6/12 |
|  |  | A407T | 1/12 |
|  |  | R409S | 1/12 |
|  |  | E419K | 1/12 |
|  |  | E428D | 1/12 |
|  |  | N432I | 1/12 |
|  |  | A457S | 1/12 |
|  |  | F463S | 1/12 |
|  |  | S465P | 1/12 |
|  |  | T470I | 1/12 |

^a^protease and gag amino acid numbering
