# Supplementary material for: Changes in codon-pair bias of human immunodeficiency virus type 1 have profound effects on virus replication in cell culture
Source: Retrovirology. 2013 Jul 25;10:78. doi: 10.1186/1742-4690-10-78 (PMC3726367; doi:10.1186/1742-4690-10-78)
Supplement: Supplementary file 5 [file 1742-4690-10-78-S2.doc]

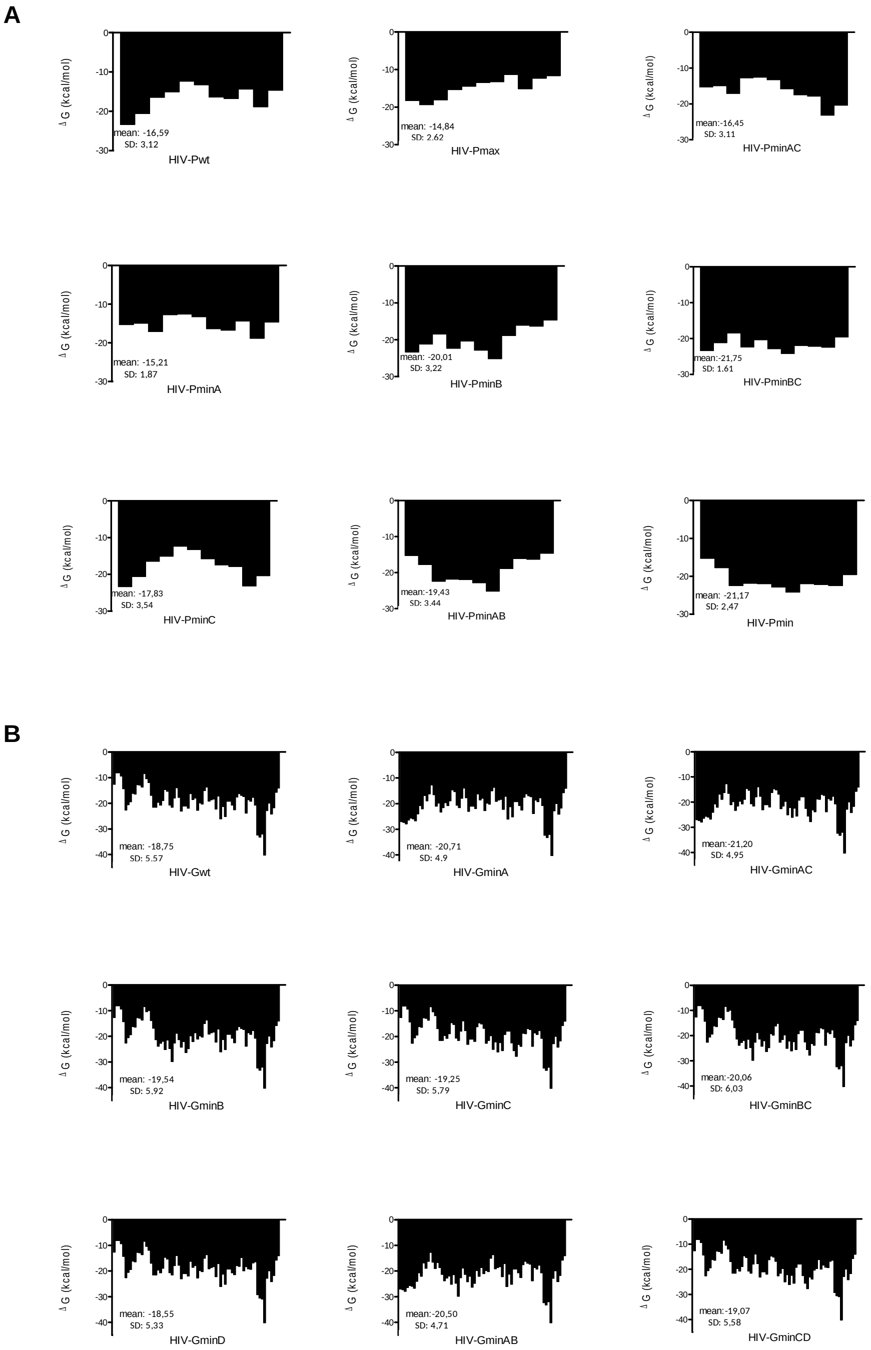
A
Δ G (kcal/mol)
mean: -16,59
SD: 3,12
HIV-Pwt
mean: -14,84
SD: 2.62
HIV-Pmax
mean:-16,45
SD: 3.11
HIV-PminAC
mean: -15,21
SD: 1,87
HIV-PminA
mean: -20,01
SD: 3,22
HIV-PminB
mean:-21,75
SD: 1,61
HIV-PminBC
mean: -17,83
SD: 3,54
HIV-PminC
mean:-19,43
SD: 3.44
HIV-PminAB
mean: -21,17
SD: 2,47
HIV-Pmin
B
mean: -18,75
SD: 5.57
HIV-Gwt
mean: -20,71
SD: 4.9
HIV-GminA
mean:-21,20
SD: 4,95
HIV-GminAC
mean: -19,54
SD: 5,92
HIV-GminB
mean: -19,25
SD: 5,79
HIV-GminC
mean:-20,06
SD: 6,03
HIV-GminBC
mean: -18,55
SD: 5,33
HIV-GminD
mean:-20,50
SD: 4,71
HIV-GminAB
mean:-19,07
SD: 5,58
HIV-GminCD

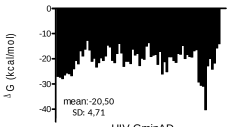

HIV-GminAD

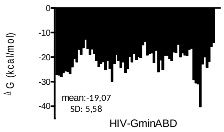

HIV-GminABD

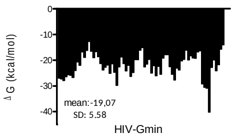

HIV-Gmin

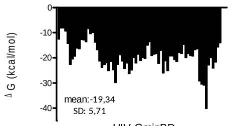

HIV-GminBD

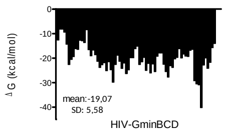

HIV-GminBCD

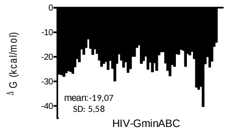

HIV-GminABC

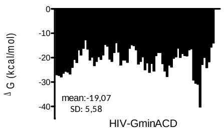

HIV-GminACD
